# Association between remnant cholesterol, metabolic syndrome, and cardiovascular disease: post hoc analysis of a prospective national cohort study

**DOI:** 10.1186/s40001-023-01369-z

**Published:** 2023-10-11

**Authors:** Junguo Jin, Xiangming Hu, Melissa Francois, Ping Zeng, Weimian Wang, Bingyan Yu, Yingling Zhou, Haojian Dong

**Affiliations:** 1 Department of Cardiology, Guangdong Cardiovascular Institute, Guangdong Provincial People’s Hospital (Guangdong Academy of Medical Sciences), Southern Medical University, Guangzhou, 510080 Guangdong China; 2 College of Medicine, SUNY Upstate Medical University, Syracuse, NY 13210 USA; 3 The Second School of Clinical Medicine, Southern Medical University, Guangzhou, 510515 Guangdong China; 4 School of Medicine, South China University of Technology, Guangzhou, 510006 China; 5 Nyingchi People’s Hospital, Nyingchi, 860000, Tibet China

**Keywords:** Remnant cholesterol, Metabolic syndrome, Inflammation, Insulin resistance, Cardiovascular disease

## Abstract

**Background:**

Epidemiologic evidence suggested that remnant cholesterol (RC) is associated with the occurrence of cardiovascular disease (CVD). In recent years, RC has been connected with different types of cardiometabolic disorders. We aim to clarify the relationship among RC, metabolic syndrome (MetS) and subsequent CVD.

**Methods:**

We enrolled 7471 individuals into our study from China Health and Nutrition Survey in 2009 and followed participants till 2015. RC was calculated as total cholesterol minus low-density lipoprotein cholesterol minus high-density lipoprotein cholesterol. CVD was defined as myocardial infarction and stroke. Multivariate logistic regression and Cox regression models were used to evaluate the association between RC and MetS as well as CVD. We further investigated whether the association between RC and CVD was mediated by MetS.

**Results:**

Of all subjects, 24.73% were diagnosed with MetS and 2.74% developed CVD. Multivariate logistic regression analysis elucidated that per-tertile-increase in RC was associated with MetS after adjusting all the confounder factors, (odds ratio: 3.49, 95% confidence interval CI 3.21–3.79, P for trend < 0.001). And per-tertile-increase RC had a significant increased risk of CVD (hazard ratio: 1.26, 95% CI 1.06–1.50, P for trend = 0.008). Meanwhile, we found that RC level is associated with the prevalence of all the components of MetS. Significant indirect effects of RC between MetS and CVD were found, with the index mediated at 48.46% of the association.

**Conclusions:**

Our study provides the evidence that RC level is independently associated with the prevalence of MetS and each component of MetS. MetS partially mediated the association between RC level and CVD risk.

## Background

According to the World Health Organization (WHO) data, cardiovascular disease (CVD) are the leading cause of death globally taking an estimated 17.9 million lives each year, with heart attacks and strokes accounting for 85% of deaths [1]. Metabolic syndrome (MetS) is a constellation of many cardio-metabolic risk factors and is associated with increased all-cause and CVD mortality risk, and as such has been arising peoples’ attention as a serious public health issue [2]. Meanwhile, the prevalence of MetS has increased in recent decades, independent of any kind of criteria for diagnosis, keeping pace with the epidemic of CVD [3]. According to previous research, the pathogenesis of MetS is not only attributable to neurohormonal activation, but also insulin resistance and chronic low-grade inflammation as well [4]. Moreover, elevated inflammatory cytokines are considered a high risk factor for the development of CVDs, and insulin resistance is regarded as one of the earliest demonstrations of CVD [5–7].

Remnant cholesterol (RC), a novel atherogenic lipoprotein, is the cholesterol content within triglyceride-rich lipoproteins, consisting primarily of very low-density lipoproteins, intermediate-density lipoproteins and chylomicron remnants. Based on a standard lipid profile, RC is usually calculated as total cholesterol minus low-density lipoprotein cholesterol (LDL-C) minus high-density lipoprotein cholesterol (HDL-C) [8]. According to a study by Johns Hopkins Medicine researchers, RC is as a stand-alone risk for CVD such as myocardial infarction and stroke [9]. Additionally, an epidemical study showed that with the level of RC increasing, the prevalence of diabetes mellitus (DM), lipid disorders and even hypertension is higher [10–13], which means that RC may act as a representative factor in a state of cardio-metabolic disorder. Interestingly, mechanistic evidence revealed that high concentrations of RC is related to low-grade inflammation and is genetically mediated by insulin resistance [14–16]. RC and MetS are linked by a positive feedback loop involving insulin resistance, chronic inflammation, abnormal lipid metabolism, and hypertension. RC affects these factors and is also affected by them, leading to faster MetS progression [17–20]. Such findings suggest significant similarities between concomitant alterations of RC and MetS in the underlying pathogenesis. Although RC was proposed as a new agent of cardiovascular risk factor, with association of many cardiometabolic disorders, the relationship between RC and MetS still unclear. Furthermore, the role of MetS played in the association between RC and CVD remains unknown.

In order to fill the knowledge gap, we investigated the correlation of RC with the prevalence of MetS and subsequent CVD based on the China Health and Nutrition Survey (CHNS). And whether the presence of MetS mediates the effect of RC on CVD.

## Methods

### Study setting and population

**Fig. 1:**
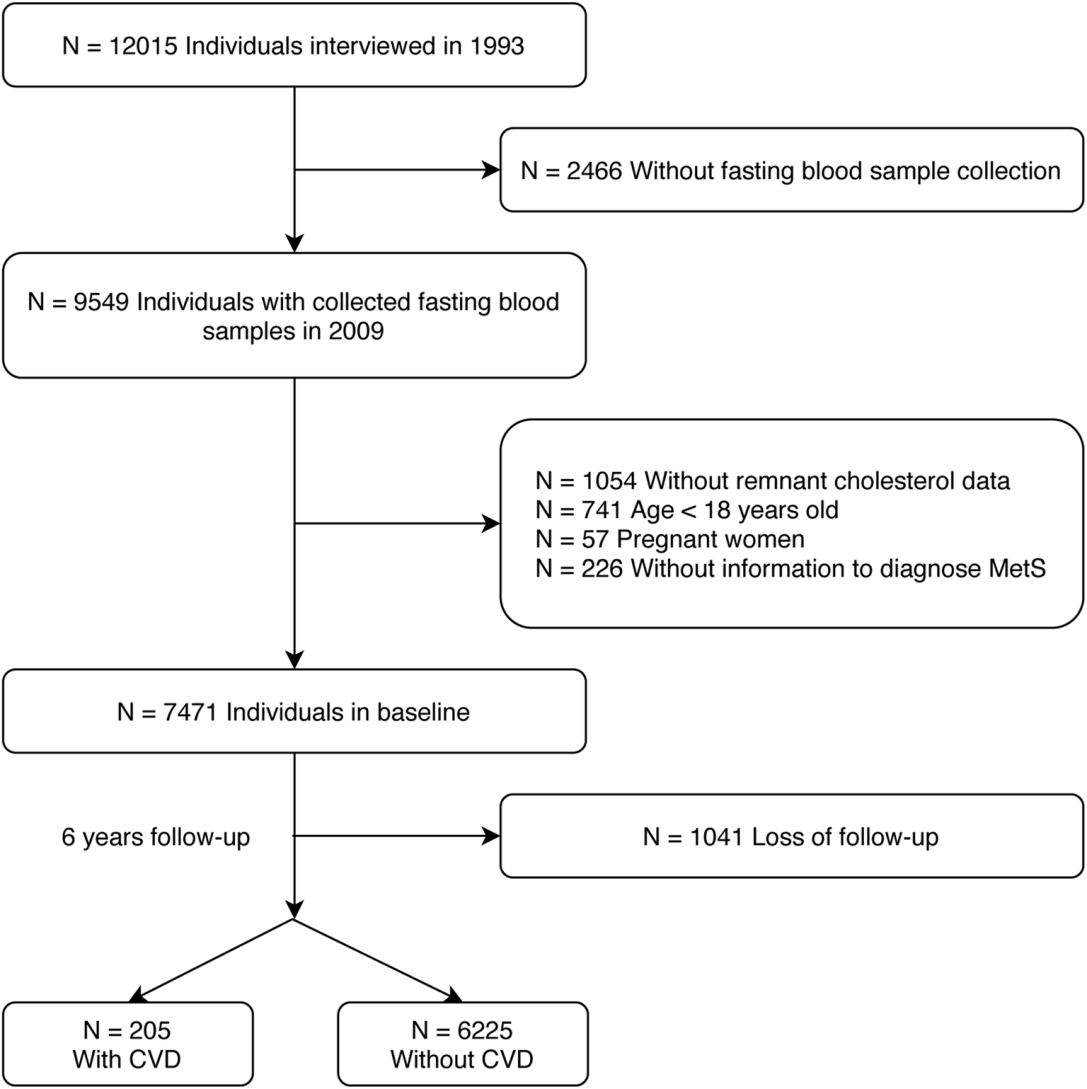
Study flowchart. *CVD* cardiovascular disease, *MetS* metabolic syndrome

This study used data from the CHNS, an ongoing longitudinal community-based cohort study carried out by the national and local governments of China. The study includes data for more than 12,000 individuals across approximately nine provinces. Trained researchers conducted household surveys, using standard questionnaires and face-to-face interviews to obtain information about participants. Each participant provided written informed consent. Study details are described in our previous article [21], and the relevant protocol is published elsewhere [22]. The inclusion criteria were: (1) age ≥ 18 years old, (2) measurement of RC level. The exclusion criteria were: (1) pregnant women, (2) without sufficient information to diagnose MetS. We first removed 2466 participants that lacked fasting blood samples, then we excluded 1054 participants without RC measurement, 741 participants who were under 18 years of age, 57 pregnant women, 226 participants without sufficient information to diagnose MetS (Fig. 1). Based on the aforementioned criteria, 7471 eligible participants were identified. We also presented demographic information, health behaviors, health status and laboratory examinations of study participants. After six years follow-up, 205 patients diagnosed with CVD, with 1041 individuals loss follow-up.

This manuscript was written in strict accordance with the STROBE statement [23].

### Measures

RC (mmol/L) was calculated as total cholesterol (TC) (mmol/L) minus LDL-C (mmol/L) minus HDL-C (mmol/L) and blood sample were obtained in the fasting state [24]. MetS was diagnosed according to the International Diabetes Federation as individuals with central obesity (waist circumference ≥ 90 cm in men or ≥ 80 cm in women) plus any two of the following: (1) raised TG (> 1.7 mmol/L) or specific treatment for TG abnormality; (2) reduced HDL-C in men (< 1.03 mmol/L) and in women (< 1.29 mmol/L) or specific treatment for TG abnormality; (3) raised blood pressure (SBP ≥ 130 mm Hg or DBP ≥ 85 mmHg) or treatment of previously diagnosed hypertension; (4) raised fasting plasma glucose (fasting plasma glucose ≥ 5.6 mmol/L) or previously diagnosed type 2 DM [25]. CVD was defined as myocardial infarction or stroke and was derived by medical diagnosis [26]from doctors.

### Definition

Height and weight were measured while the subjects were wearing light clothing and standing without shoes. We calculated body mass index (BMI) as weight (kg)/height (m)^2^. Health behaviors (smoking and alcohol consumption), education background and residence were self-reported. Smoking was defined as any previous smoking (yes/no), and alcohol consumption was defined as imbibition greater than three times per week (yes/no). Renal function was presented as eGFR using the Chronic Kidney Disease Epidemiology Collaboration (CKD-EPI) equation [27]. Nutrition intake was assessed through questionnaire that included 24 h diet recalls about the food on the same 3 days (2 week days and 1 weekend day). Energy intake, carbohydrate intake, fat intake and protein intake were calculated by multiplying the intake of each food from the average dietary intake for 3 days by the standard serving size (100 g). Physical activity was defined as more than 150 min of moderate exercise or more than 75 min of vigorous exercise per week [28]. Creatinine, uric acid, fasting blood glucose, HDL-C, LDL-C, TG, TC,were measured using a Hitachi 7600 machine (Randox, UK and Kyowa, Japan). HbA1c was detected by HLC-723 G7/D10/PDQ A1c (Tosoh, Japan/Bio-Rad, USA/Primus, USA). Insulin was detected by Gamma counter XH-6020,China (North Institute of Bio-Tech,China). High-sensitivity C-reactive protein (Hs-CRP) was measured by Hitachi 7600 machine (Denka Seiken, Japan).

Homeostatic model assessment of insulin resistance (HOMA-IR) was calculated by: fasting insulin (µIU/mL) × fasting glucose (mmol/L)/22.5.

### Statistical methods

Participant characteristics were described based on tertiles of RC. Continuous variables are expressed as means ± standard deviation for normal distributions or medians and interquartile range (25% to 75%) for skewed distributions. Categorical variables are presented as relative frequencies (percentages). Each tertile of RC was taken as a unit and p-values for trends were calculated using linear-regression analyses for continuous variables and Cochran-Armitage test for categorical variables. We used an upset and correlation plot to show the distribution of different combinations of MetS components and the relationships between the MetS components. Generalized additive models were then used to identify relationships between RC and the prevalence of each MetS component, since RC was a continuous variable. The independent association of RC with MetS and CVD were evaluated using logistic models with odds ratios (ORs) and 95% confidence intervals (CIs), and Cox regression models with hazard ratios (HRs) and 95% CIs. Potential covariates that were significant in the baseline comparison, or that we considered to be of clinical importance were included in the multivariate models. We established two main models for covariate adjustment: crude model; adjusted model for age, sex, education, residence, smoking, alcohol consumption, protein intake, carbohydrate intake, fat intake, daily energy intake and physical activity. Subgroup analyses and effect modification were performed considering age (< 60/ ≥ 60 years old), sex (male/female), education levels (middle school and below/high school and above), alcohol consumption (yes/no), smoking(yes/no) and residence(urban/rural). Finally, given that the MetS has been identified as a way through which RC may affect the CVD, we investigated whether the association between RC and CVD was mediated by MetS. The bootstrap test was used to assess the effects of these mediators [29].

The sensitivity analysis was conducted by adopting the WHO criterion to diagnose MetS [26]. The proportion of missing data in the analytic sample did not exceed 2%. Missing data were interpolated using the method of last observation carried forward or using the means and medians for continuous variables and skewed variables. Comparisons where P was < 0.05 (two-sided) were considered to be statistically significant. We performed all analyses with Stata 15.0, R (version 3.4.3) and EmpowerStats (http://www.empowerstats.com, X and Y Solutions, Inc., Boston, MA).

## Results

### Baseline information

Demographic characteristics of the study population are summarized in Tables 1 and 2. Among the 7471 participants, there were 1848 individuals (24.74%) who had MetS and 205 individuals (2.74%) with CVD. Urban residents accounted for the majority of subjects across the different RC groups. Subjects with MetS exhibited statistically higher RC levels and had significantly higher BMI, uric acid, creatinine, LDL-C, TG, TC, HOMA-IR, Hb1Ac, fasting blood glucose, and insulin levels. Conversely eGFR and HDL-C levels were significantly lower within the elevated RC tertile. Compared with lower-level RC group, subjects within higher tertiles of RC were older, more educated, and engaged in smoking and alcohol consumption more frequently, and their protein intake was also higher. Participants were spilt into two groups, according to whether MetS was present. Female and urban residents accounted for the majority of subjects in the group with MetS. These subjects were statistically significant higher in age, BMI, uric acid, Hs-CRP, LDL-C, TG, TC, RC, HOMA-IR, HbA1c, fasting blood glucose, carbohydrate intake, daily energy intake and insulin levels. Subjects with MetS also had statistically significantly lower HDL-C and eGFR when compared to participants without MetS.

**Table 1:** Baseline information according to tertiles of RC

RC	Q1 (< 0.25) *n* = 2457	Q2 (0.25–0.52) *n* = 2464	Q3 (> 0.52) *n* = 2550	*P* for trend
Age (years)	50 ± 15	51 ± 16	51 ± 14	< 0.001
Male sex	1071 (43.59%)	1134 (46.02%)	1360 (53.33%)	< 0.001
BMI (kg/m^2^)	22.43 ± 3.16	23.27 ± 3.43	24.69 ± 3.52	< 0.001
Residence				0.006
Urban	1692 (68.86%)	1642 (66.64%)	1662 (65.18%)	
Rural	765 (31.14%)	822 (33.36%)	888 (34.82%)	
High school and above	542 (22.06%)	600 (24.35%)	640 (25.10%)	0.012
Smoking	708 (28.82%)	739 (29.99%)	908 (35.61%)	< 0.001
Alcohol consumption	660 (26.86%)	634 (25.73%)	805 (31.57%)	< 0.001
Uric acid (μmol/L)	274.64 ± 79.84	298.73 ± 84.03	364.08 ± 129.64	< 0.001
Hs-CRP (mg/L)	1.00 (0.00–2.00)	1.00 (0.00–2.00)	2.00 (1.00–3.00)	< 0.001
Creatinine (μmol/L)	85.81 ± 16.44	88.82 ± 30.71	89.01 ± 16.65	< 0.001
eGFR (ml/min/m^2^)	80.38 ± 16.84	78.47 ± 17.31	78.26 ± 16.54	< 0.001
HDL-C (mmol/L)	1.57 ± 0.34	1.41 ± 0.32	1.19 ± 0.30	< 0.001
LDL-C (mmol/L)	2.97 ± 0.88	2.99 ± 0.93	2.83 ± 0.98	< 0.001
TG (mmol/L)	0.93 ± 0.50	1.35 ± 0.67	2.98 ± 1.94	< 0.001
TC (mmol/L)	4.68 ± 0.94	4.77 ± 0.98	5.11 ± 1.04	< 0.001
RC (mmol/L)	0.14 (0.08–0.19)	0.36 (0.30–0.43)	0.85 (0.65–1.21)	< 0.001
HOMA-IR	2.87 ± 6.27	3.44 ± 5.67	5.35 ± 10.07	< 0.001
HbA1c (%)	5.50 ± 0.72	5.59 ± 0.84	5.78 ± 1.06	< 0.001
Fasting blood glucose (mmol/L)	5.12 ± 1.03	5.31 ± 1.24	5.87 ± 1.98	< 0.001
Insulin (uIU/mL)	11.85 ± 20.59	13.71 ± 18.27	18.64 ± 28.99	< 0.001
Protein intake (g)	61.91(49.44–77.28)	61.95(49.91–77.39)	63.73(50.77–79.44)	0.004
Carbohydrate intake (g)	296.92 ± 104.20	291.95 ± 98.92	291.54 ± 99.86	0.113
Fat intake (g)	67.70(48.23–992.26)	70.10(50.06–94.01)	70.58(49.96–95.19)	0.052
Daily energy intake (kcal)	2124.48 ± 654.85	2125.74 ± 658.21	2142.24 ± 669.24	0.567
Physical activity	1716(69.8%)	1710(69.4%)	1711(67.1%)	0.079
MetS	214 (8.71%)	454 (18.43%)	1180 (46.27%)	< 0.001
Abdominal obesity	856 (34.84%)	1073 (43.55%)	1432 (56.16%)	< 0.001
Elevated triglyceride	91 (3.70%)	429 (17.41%)	2105 (82.55%)	< 0.001
Reduced HDL-C	256 (10.42%)	550 (22.32%)	1174 (46.04%)	< 0.001
Raised blood pressure	870 (35.41%)	1009 (40.95%)	1271 (49.84%)	< 0.001
Abnormal glucose metabolism	470 (19.13%)	624 (25.32%)	1021 (40.04%)	< 0.001

*BMI* body mass index, *eGFR* estimated glomerular filtration rate, *HbA1c* hemoglobin A1c, *HDL-C* high-density lipoprotein cholesterol, *HOMA-IR* homeostatic model assessment for insulin resistance, *Hs-CRP* high-sensitivity C-reactive protein, *LDL-C* low-density lipoprotein cholesterol, *MetS* metabolic syndrome, *RC* remnant cholesterol, *TC* total cholesterol, *TG* triglyceride

**Table 2:** Baseline information according to whether MetS existed

	Non-MetS *n* = 5623	MetS *n* = 1848	*P*-value
Age (years)	48.99 ± 15.35	55.90 ± 12.90	< 0.001
Male sex	2873 (51.09%)	692 (37.45%)	< 0.001
BMI (kg/m^2^)	22.52 ± 3.02	26.40 ± 3.24	< 0.001
Residence			0.027
Urban	3799 (67.56%)	1197 (64.77%)	
Rural	1824 (32.44%)	651 (35.23%)	
High school and above	1428 (25.40%)	354 (19.16%)	< 0.001
Smoking	1885 (33.52%)	470 (25.43%)	< 0.001
Alcohol consumption	1664 (29.59%)	435 (23.54%)	< 0.001
Uric acid (μmol/L)	301.41 ± 100.80	348.72 ± 119.59	< 0.001
Hs-CRP (mg/L)	1.00 (0.00–2.00)	2.00 (1.00–4.00)	< 0.001
Creatinine (μmol/L)	88.04 ± 23.67	87.45 ± 17.42	0.308
eGFR (ml/min/m^2^)	80.52 ± 16.84	74.46 ± 16.36	< 0.001
HDL-C (mmol/L)	1.45 ± 0.35	1.21 ± 0.31	< 0.001
LDL-C (mmol/L)	2.87 ± 0.89	3.11 ± 1.04	< 0.001
TG (mmol/L)	1.45 ± 1.13	2.74 ± 2.03	< 0.001
TC (mmol/L)	4.74 ± 0.96	5.21 ± 1.05	< 0.001
RC (mmol/L)	0.30 (0.17–0.52)	0.68 (0.40–1.10)	< 0.001
HOMA-IR	2.20 (1.53–3.20)	3.62 (2.38–6.05)	< 0.001
HbA1c (%)	5.48 ± 0.72	6.07 ± 1.18	< 0.001
Fasting blood glucose (mmol/L)	5.17 ± 1.14	6.26 ± 2.09	< 0.001
Insulin (uIU/ml)	12.76 ± 18.52	20.92 ± 33.30	< 0.001
Protein intake (g)	62.82(50.32–78.07)	62.07(49.40–78.24)	0.327
Carbohydrate intake (g)	296.78 ± 101.62	283.29 ± 98.51	< 0.001
Fat intake (g)	69.45(49.49–93.75)	69.11(49.02–94.09)	0.572
Daily energy intake (kcal)	2145.06 ± 655.56	2088.06 ± 671.25	0.001
Physical activity	3882(69.0%)	1255(67.9%)	0.380
MetS	214 (8.71%)	454 (18.43%)	< 0.001
Abdominal obesity	1513 (26.91%)	1848 (100.00%)	< 0.001
Elevated triglyceride	1264 (22.48%)	1361 (73.65%)	< 0.001
Reduced HDL-C	946 (16.82%)	1034 (55.95%)	< 0.001
Raised blood pressure	1784 (31.73%)	1366 (73.92%)	< 0.001
Abnormal glucose metabolism	1004 (17.86%)	1111 (60.12%)	< 0.001

*BMI* body mass index, *eGFR* estimated glomerular filtration rate, *HbA1c* hemoglobin A1c, *HDL-C* high-density lipoprotein cholesterol, *HOMA-IR* homeostatic model assessment for insulin resistance, *Hs-CRP* high-sensitivity C-reactive protein, *LDL-C* low-density lipoprotein cholesterol, *MetS* metabolic syndrome, *RC* remnant cholesterol, *TC* total cholesterol, *TG* triglyceride

### The intersection distribution of different metabolic disorders

**Fig. 2:**
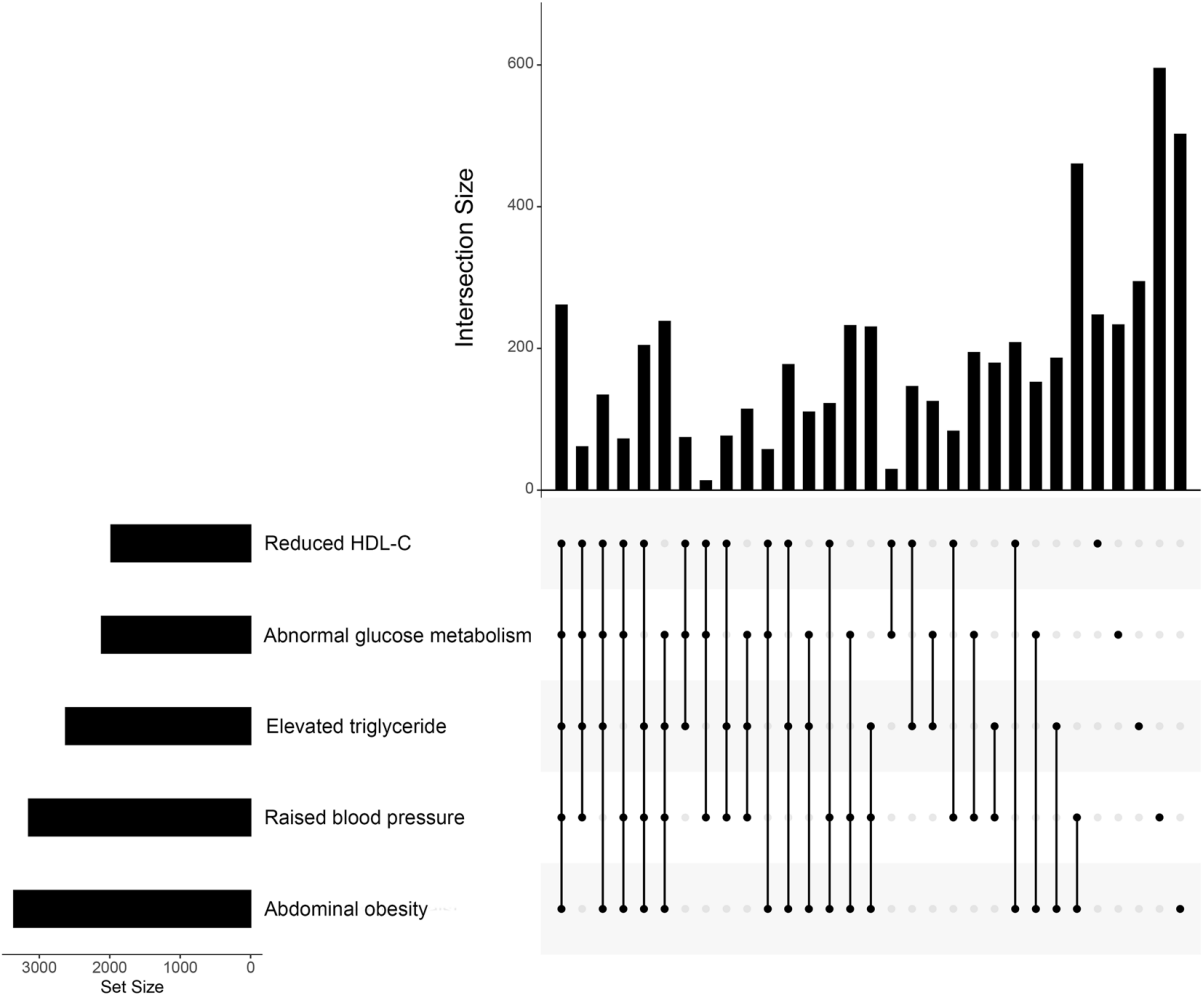
The upset plot of the intersection distribution of different metabolic disorders. *HDL-C* high-density lipoprotein cholesterol

By analyzing the upset plot (Fig. 2), we observed that among the general metabolic disorders, individuals with expanded waist circumference were the most prevalent in the general population, and participants with low HDL-C were relatively rare by contrast. Corresponding to the diagnosis of MetS, the population with all five factors coexisting accounted for the majority of MetS cases, while the proportion of the participants with the combination of expanded waist circumference, high blood glucose and low HDL-C was comparatively small.

### The association of RC and MetS

**Fig. 3:**
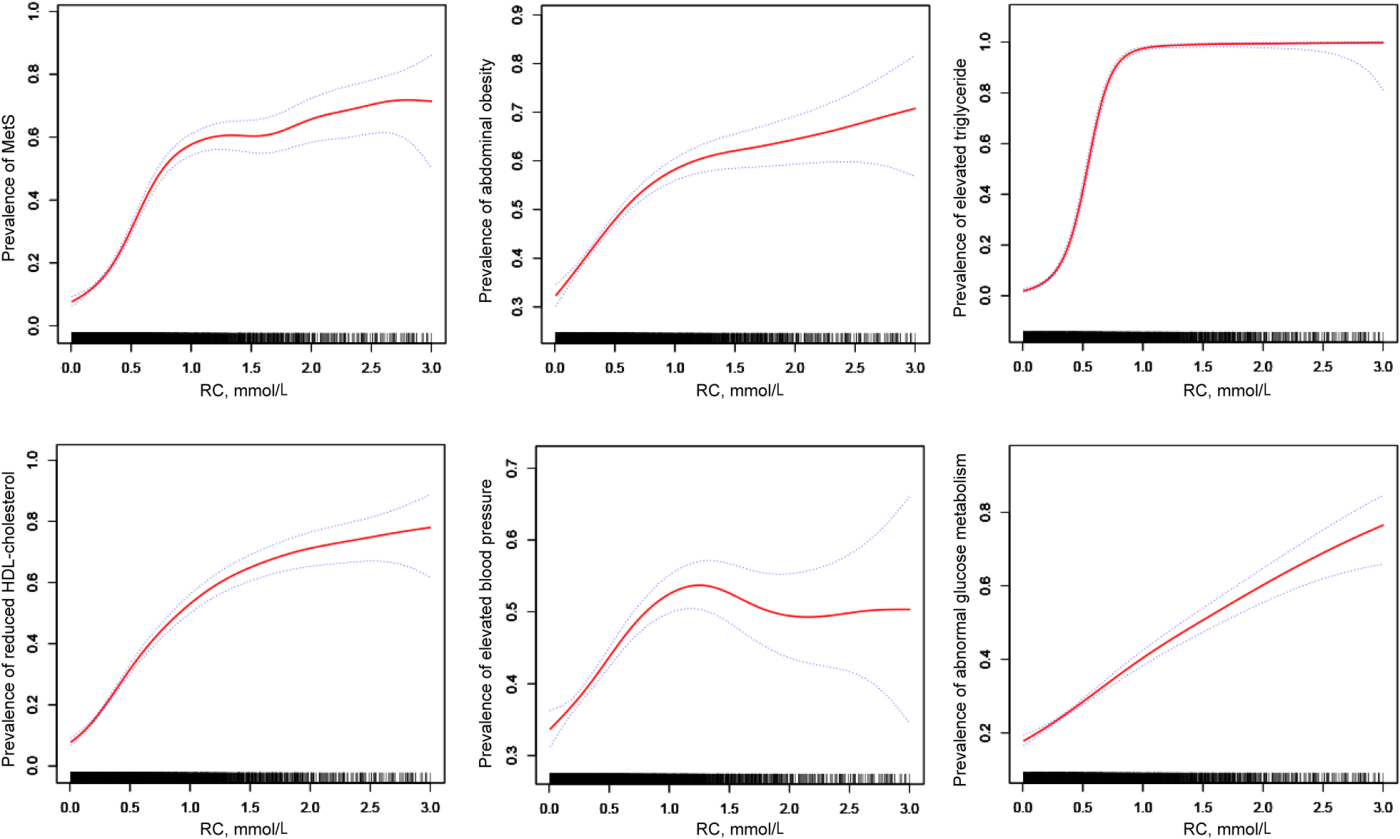
The association of RC and *MetS* and metabolic disorders, *RC* remnant cholesterol; *MetS* metabolic syndrome

**Fig. 4:**
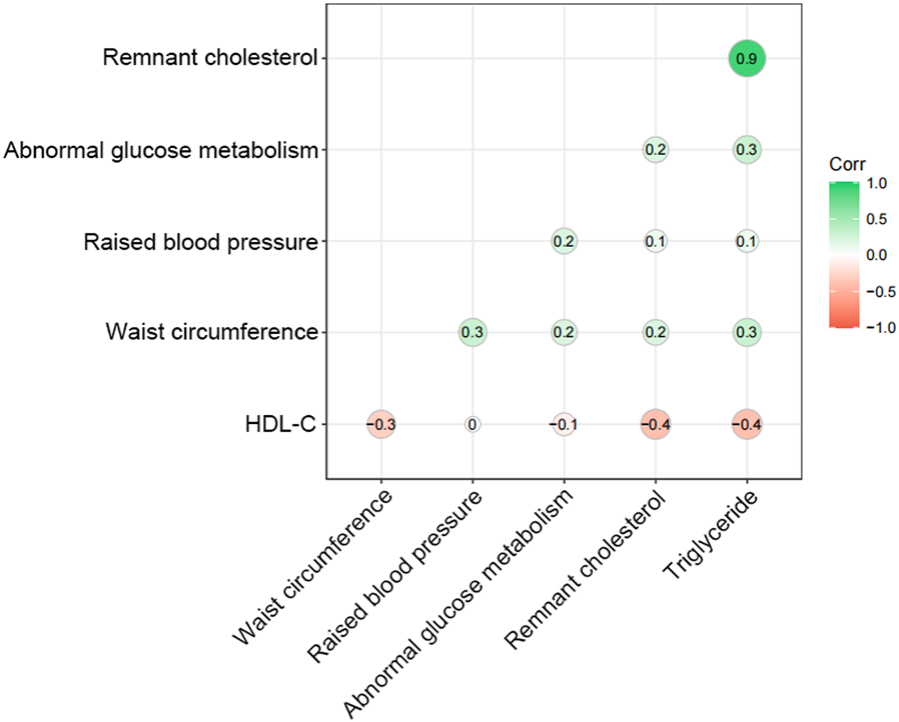
The correlation between RC and the component of MetS. *HDL-C* high-density lipoprotein cholesterol

Table 3 shows the association between RC tertiles and prevalence of MetS. By using logistic regression models, per-tertile-increase in RC were associated with 317% increasing risk for MetS (OR: 3.17, 95% CI 2.93–3.43, *P* for trend < 0.001). After adjusting for age, sex, education, residence, alcohol consumption, protein intake, carbohydrate intake, fat intake, daily energy intake and physical activity, the result remained significant (OR: 3.49, 95% CI 3.21–3.79, *P* for trend < 0.001). As shown in Fig. 3, with rising concentration of RC, the prevalence of MetS, abdominal obesity, elevated triglycerides, reduced HDL-C, elevated blood pressure, and abnormal glucose metabolism simultaneously increased. After analyzing the correlation between RC and each component of MetS in Fig. 4, we found out that RC level had a positive correlation with waist circumference, abnormal glucose metabolism, raised blood pressure and triglyceride levels, with the triglycerides serving as the most relevant factor for RC. All components were correlated with each other positively except for reduced HDL-C, which has negative correlation with RC, triglyceride, abnormal glucose metabolism and waist circumference. Though, HDL-C is irrelevant to raised blood pressure.

**Table 3:** OR (95% CIs) for MetS of different tertiles of RC and covariates

Variable	Univariate OR (95% CI)	*P*-value	Multivariate OR (95% CI)	*P*-value
RC				
Q1 (< 0.25)	Ref.	–	Ref.	–
Q2 (0.25–0.52)	2.37 (1.99–2.82)	< 0.001	2.41 (2.02–2.88))	< 0.001
Q3 (> 0.52)	9.03 (7.71–10.62)	< 0.001	10.72 (9.08–12.71)	< 0.001
Each 1 tertile increase in RC	3.17 (2.93–3.43)	< 0.001	3.49 (3.21–3.79)	< 0.001
Age (years)	1.03 (1.03–1.04)	< 0.001	1.04 (1.03–1.04)	< 0.001
Sex	0.57 (0.51–0.64)	< 0.001	0.45 (0.38–0.53)	< 0.001
Education	0.70 (0.61–0.79)	0.001	0.81 (0.69–0.96)	0.012
Rural residence	1.17 (1.01–1.34)	0.027	0.98 (0.86–1.12)	0.794
Smoking	0.68 (0.60–0.76)	< 0.001	0.85 (0.72–1.01)	0.064
Drinking	0.73 (0.65–0.83)	< 0.001	1.14 (0.96–1.34)	0.130
Protein intake (g)	1.00 (1.00–1.00)	0.530	1.01 (1.00–1.01)	< 0.001
Carbohydrate intake (g)	1.00 (1.00–1.00)	< 0.001	1.00 (1.00–1.00)	0.989
Fat intake (g)	1.00 (1.00–1.00)	0.810	1.00 (1.00–1.01)	0.692
Daily energy intake (kcal)	1.00 (1.00–1.00)	0.001	1.00 (1.00–1.00)	0.652
Physical activity	0.95 (0.85–1.06)	0.365	0.89 (0.77–1.13)	0.109

Data were shown as OR (95%CI) which was evaluated using logistic regression models

*OR* odd ratio, *RC* remnant cholesterol, *95% CI* 95% confidence interval

### The association of RC and new-onset CVD

After a median follow-up of 6 years, we identified 205 (2.74%) subjects who developed CVD. By using the Cox regression analysis in Table 4, RC had a significant correlation with risk of CVD in crude model (HR: 1.22, 95% CI 1.03–1.45, *P* for trend = 0.021). After adjusting for age, sex, education, residence, alcohol consumption, protein intake, carbohydrate intake, fat intake, daily energy intake and physical activity, the result remained significant (HR: 1.26, 95% CI 1.06–1.50, *P* for trend = 0.008).

**Table 4:** HR (95% CIs) for CVD of different tertiles of RC and covariates

Variable	Univariate HR (95% CI)	*P*-value	Multivariate HR (95% CI)	*P*-value
RC				
Q1 (< 0.25)	Ref.		Ref.	
Q2 (0.25–0.52)	1.38 (0.97–1.97)	0.078	1.34 (0.94–1.92)	0.108
Q3 (> 0.52)	1.52 (1.07–2.15)	0.019	1.61 (1.13–2.28)	0.008
Each 1 tertile increase in RC	1.22 (1.03–1.45)	0.021	1.26 (1.06–1.50)	0.008
Age (years)	1.07 (1.06–1.08)	< 0.001	1.07 (1.05–1.08)	< 0.001
Sex	1.28 (0.98–1.69)	0.075	1.74 (1.22–2.47)	0.002
Education	0.56 (0.38–0.83)	0.004	0.85 (0.55–1.32)	0.466
Rural residence	1.28 (0.96–1.70)	0.092	1.38 (1.01–1.88)	0.042
Smoking	1.06 (0.80–1.42)	0.681	0.87 (0.61–1.24)	0.447
Drinking	0.63 (0.45–0.89)	0.008	0.59 (0.40–0.87)	0.008
Protein intake (g)	0.99 (0.98–1.00)	0.004	0.99 (0.98–1.00)	0.032
Carbohydrate intake (g)	1.00 (1.00–1.00)	0.286	1.00 (0.99–1.01)	0.880
Fat intake (g)	1.00 (1.00–1.00)	0.610	1.00 (1.00–1.00)	0.651
Daily energy intake (kcal)	1.00 (1.00–1.00)	0.454	1.00 (1.00–1.00)	0.402
Physical activity	1.57 (1.12–2.20)	0.009	1.40 (0.97–2.02)	0.071

Data were shown as HR (95%CI) which was evaluated using Cox regression models

*HR* hazard ratio, *RC* remnant cholesterol, *95% CI* 95% confidence interval

### Subgroup analysis

Subgroup analyses of the asocciation between RC, MetS and CVD was listed in Table 5 and Table 6. There was an interaction between age and RC for MetS, as people under 60 years old at high RC levels were significantly associated with MetS (OR: 3.84, 95%CI 3.45–4.29, *P* for interaction < 0.001). However, other stratification factors such as sex, education, alcohol consumption, smoking and residence had no interaction with RC regarding the incidence of metabolic syndrome. No interaction were found of all subgroups in the relationship between RC and CVD.

**Table 5:** The subgroup analysis between RC and MetS

Variables	No. of participants	OR (95% CI)	*P*-value	*P* for interaction
Age				< 0.001
< 60	5326	3.84 (3.45–4.29)	< 0.001	
≥ 60	2145	2.77 (2.43–3.17)	< 0.001	
Sex				0.588
Male	3565	3.38 (2.96–3.88)	< 0.001	
Female	3906	3.50 (3.15–3.89)	< 0.001	
Education				0.568
Middle school and below	5689	3.48 (3.17–3.82)	< 0.001	
High school and above	1782	3.44 (2.85–4.18)	< 0.001	
Alcohol consumption				0.083
Yes	5372	3.14 (2.67–3.71)	< 0.001	
No	2099	3.60 (3.28–3.97)	< 0.001	
Smoking				0.288
Yes	2355	3.77 (3.18–4.50)	< 0.001	
No	5116	3.37 (3.07–3.71)	< 0.001	
Residence				0.823
Urban	4996	3.51 (3.17–3.89)	< 0.001	
Rural	2475	3.47 (3.01–4.01)	< 0.001	

*OR* odd ratio, *95% CI* 95% confidence interval

**Table 6:** The subgroup analysis between RC and CVD

Variables	No. of participants	HR (95% CI)	*P-*value	*P* for interaction
Age				0.216
< 60	5326	1.11 (0.85–1.47)	0.437	
≥ 60	2145	1.35 (1.09–1.68)	0.007	
Sex				0.347
Male	3565	1.18 (0.93–1.49)	0.168	
Female	3906	1.37 (1.06–1.77)	0.015	
Education				0.369
Middle school and below	5689	1.29 (1.07–1.55)	0.007	
High school and above	1782	1.00 (0.63–1.60)	0.992	
Alcohol consumption				0.24
Yes	5372	1.05 (0.73–1.51)	0.796	
No	2099	1.32 (1.09–1.61)	0.005	
Smoking				0.193
Yes	2355	1.25 (0.93–1.69)	0.146	
No	5116	1.24 (1.01–1.51)	0.037	
Residence				0.096
Urban	4996	1.38 (1.12–1.72)	0.003	
Rural	2475	1.04 (0.79–1.38)	0.778	

*HR* hazard ratio, *95% CI* 95% confidence interval

### Mediation analysis

**Fig. 5:**
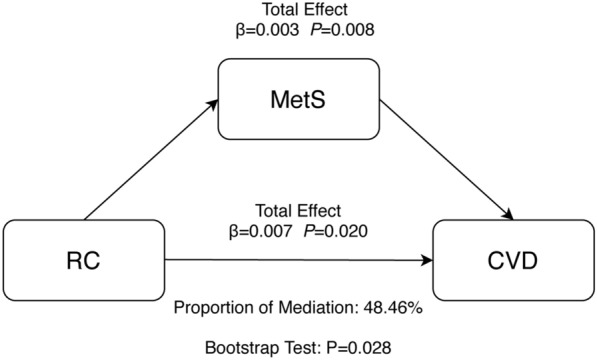
Mediation analysis of the association between RC and CVD. *CVD* Cardiovascular disease, *MetS* Metabolic syndrome, *RC* Remnant cholesterol

Potential mediation effects of RC in the correlation between MetS and CVD are presented in Fig. 5. We observed significant indirect effects of RC between MetS and CVD with mediation at 48.46% of the association.

### Sensitivity analysis

**Fig. 6:**
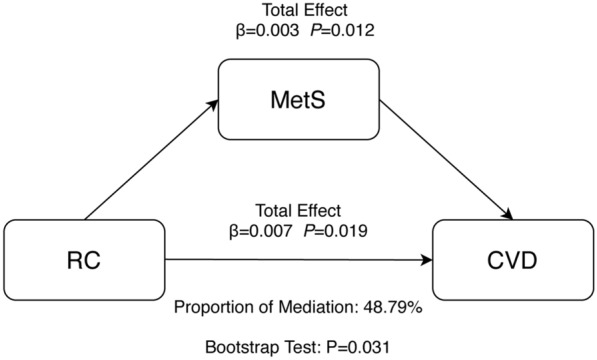
Mediation analysis of the association between RC and CVD (WHO criterion for MetS). *CVD* Cardiovascular disease, *MetS* Metabolic syndrome, *RC* Remnant cholesterol

To consolidate our result, we conducted the sensitivity analysis base on the different diagnostic criterion of MetS (WHO standard) and the results were listed below in Table 7 and Fig. 6. We re-did the mediation analysis and logistic regression. The results were similar to the main results.

**Table 7:** OR (95% CIs) for MetS (WHO criterion) of different tertiles of RC and covariates

Variable	Univariate OR (95% CI)	*P*-value	Multivariate OR (95% CI)	*P*-value
RC				
Q1 (< 0.25)	Ref.		Ref.	
Q2 (0.25–0.52)	2.52 (2.11–3.02)	< 0.001	2.54 (2.12–3.05)	< 0.001
Q3 (> 0.52)	8.15 (6.92–9.65)	< 0.001	8.86 (7.49–10.53)	< 0.001
Each 1 tertile increase in RC	2.94 (2.72–3.18)	< 0.001	3.07 (2.84–3.35)	< 0.001
Age (years)	1.03 (1.03–1.04)	< 0.001	1.04 (1.03–1.04)	< 0.001
Sex	0.80 (0.72–0.89)	< 0.001	0.65 (0.55–0.77)	< 0.001
Education	0.73 (0.64–0.83)	< 0.001	0.89 (0.75–1.05)	0.157
Rural residence	1.00 (0.89–1.12)	0.944	0.86 (0.75–0.98)	0.028
Smoking	0.91 (0.81–1.03)	0.122	0.98 (0.83–0.16)	0.832
Drinking	0.92 (0.81–1.04)	0.178	1.17 (0.99–1.38)	0.060
Protein intake (g)	1.00 (1.00–1.00)	0.450	1.01 (1.00–1.01)	0.012
Carbohydrate intake (g)	1.00 (1.00–1.00)	< 0.001	1.00 (1.00–1.00)	0.778
Fat intake (g)	1.00 (1.00–1.00)	0.729	1.00 (0.99–1.01)	0.939
Daily energy intake (kcal)	1.00 (1.00–1.00)	0.0136	1.00 (1.00–1.00)	0.888
Physical activity	1.05 (0.94–1.18)	0.397	0.98 (0.85–1.13)	0.805

WHO criterion: insulin resistance (defined as type 2 DM or impaired fasting glucose (> 100 mg/dl) or impaired glucose tolerance) plus two of the following: (1) abdominal obesity (waist-to-hip ratio > 0.9 in men or > 0.85 in women, or BMI > 30 kg/m^2^, (2) triglycerides ≥ 150 mg/dL, and/or HDL-C < 40 mg/dL in men or < 50 mg/dL in women, (3) systolic blood pressure/diastolic blood pressure ≥ 140/90 mmHg, (4) microalbuminuria (urinary albumin secretion rate ≥ 20 μg/min, or albumin-to-creatinine ratio ≥ 30 mg/g). Data were shown as OR (95%CI) which was evaluated using logistic regression models

*OR* odd ratio, *RC* remnant cholesterol, *95% CI* 95% confidence interval

## Discussion

In this nationwide study, we found that RC was significantly associated with the prevalence of MetS and incidence of CVD. RC was also positively related with the each component of MetS, which was most relevant to elevated triglycerides and has minimal correlation with raised blood pressure. Additionally, MetS partially mediates the relationships between RC and CVD.

A remarkable increase in the prevalence of obesity is garnering greater the attention from the public [30]. Obesity is a predominant contributor to MetS, and the presence of the obesity elevates the risk of dyslipidemia by raising the concentration of TG levels and lowering concentration of the HDL-C [31, 32]. In the present study, with increasing tertile of RC, the prevalence of elevated triglycerides and reduced HDL-C were higher, which was consistent with previous studies. Besides, the correlation between RC and TG was the highest compared to RC and other components of MetS, which was in line with previous research which pointed out that TG was primarily carried by the remnants and the concentration of TG was highly increased with the elevated levels of RC [12, 33]. At the same time, levels of the HDL-C decreased as levels of the RC increased because of the exchange of the TGs and cholesterol between the HDL-C and remnants in plasma [11, 34]. The reverse correlation between the HDL-C and RC was in line with our results. Previous epidemiological studies found that elevated RC along with a higher prevalence of DM, as such RC may be a predictor of diabetes or prediabetes and hypertension [35, 36]. Additionally, a study of a population of 7308 individuals recruited from CHNS showed that the concentration of RC was associated with DM beyond the LDL-C [37]. Besides, RC also had a substantial connection with the prediction or diagnosis of hypertension, and could be used as a blood marker for screening [38, 39]. A recent study, which included more than 8 million Korean adults, found that RC provide additional information in predicting future progression of type 2 DM, independent of the conventional lipid parameters [40]. All of the above evidence indicated that RC level was highly relevant to cardiometabolic disorders.

Furthermore, our study found that MetS mediated the association between RC and new-onset CVD. Previous study found that RC levels of ≥ 1 mmol/L (39 mg/dL) was presented in 22% of the population, which was associated with a two-fold increase in mortality from cardiovascular and other causes [41]. High levels of both RC and LDL-C were associated with a higher risk of CVD than either one indicator alone [42]. Elevated RC levels, independent of LDL-C levels, were also associated with an increased risk of incident CVD. Thses findings were novel and similar to our findings. In recent years, RC had attracted a lot of attention as a residual cardiovascular risk factor in many large cohort studies, and was highly instrumental due to its great atherogenic capacity [43, 44]. Unlike LDL-C, RC is free to enter the intima and become trapped in the connective tissue matrix, and could also be taken up by macrophages without modification. Furthermore, it is difficult for RC to diffuse back to blood stream, since RC has larger scales compared to LDL-C [45, 46]. Our study suggested that the association between RC and CVD may be related not only to RC causing systemic low-level inflammation and insulin resistance, but also due to RC causing a series of abnormal manifestations of cardiovascular metabolism represented by MetS which accelerated the development of CVD. However, more studies are required to support this mechanistic correlation.

Therefore, the monitoring of RC, as a simple method, could further facilitate the prevention of CVD. Special attention needs to be paid to the cardiometabolic disorders when we studied RC, in order to early identify its related metabolic risks. Future studies are needed to provide evidence that whether a reduction in RC could reduce the risk of CVD by improving metabolic status.

## Limitations

Several limitations of the current study should be taken into consideration. First, due to the nature of cross-sectional design of the current study, we can only determine the associations between RC and MetS, rather than causality. Future prospective studies are needed to identify the mechanism by which elevated RC is associated with increased risk of MetS. Second, in the current study we calculated the concentration of RC from a standard lipid profile as total cholesterol minus LDL-C minus HDL-C, which was not as accurate as direct approaches like ultracentrifugation or nuclear magnetic resonance spectroscopy. However, owing to the side effects of using such laborious methods like cost and time-consumption, it is not appropriate for their use to be widespread. Third, although our analysis utilized different approaches to support our discovery, we must be cautious when explaining these results as some observed associations may be accidental. For instance, family history of CVD and other chronic diseases may influence the current findings, but this information was not provided by CHNS. Finally, repeated analysis across different ethnic groups may enhance the extrapolation of conclusions.

## Conclusions

In summary, our study provided the evidence that RC level was independently associated with the prevalence of MetS and incidence of CVD, and was also primarily connected with each component of MetS. Insulin resistance and inflammation need to be considered as intermediate pivotal links between RC, MetS and CVD. Ultimately, MetS partially mediated the association between RC level and CVD risk, and the intrinsic mechanism by which this occurs needs further investigation.

## Data Availability

The data analyzed in this study can be available at: https://www.cpc.unc.edu/projects/china.

## Abbreviations

BMI: Body mass index
CHNS: China Health and Nutrition Survey
CVD: Cardiovascular disease
DM: Diabetes mellitus
eGFR: Estimated glomerular filtration rate
HbA1c: Hemoglobin A1c
HDL-C: High-density lipoprotein cholesterol
HOMA-IR: Homeostatic model assessment for insulin resistance
Hs-CRP: High-sensitivity C-reactive protein
LDL-C: Low-density lipoprotein cholesterol
MetS: Metabolic syndrome
OR: Odds ratio
RC: Remnant cholesterol
TC: Total cholesterol
TG: Triglyceride
95% CI: 95% confidence interval
